# A Case of Almost Painless Herpes Zoster Presenting with Symptoms of Cystitis, Penile Numbness, and Acute Vestibular Failure

**DOI:** 10.1155/2013/738579

**Published:** 2013-10-22

**Authors:** Hussain Al-Sardar

**Affiliations:** Department of Medicine, Southend University Hospital, Prittlewell Chase, Westcliff-on-Sea, Essex SS0 0RY, UK

## Abstract

Herpes zoster (shingles) is an acute, painful, vesicular, and cutaneous eruption caused by varicella zoster virus, the same virus which causes chicken pox. It is due to the reactivation of the virus which remains dormant in sensory ganglions following chicken pox. It is usually confined to a single dermatome but may involve 2-3 dermatomes. Typically, it is a unilateral lesion which can affect both cranial and peripheral nerves. It is usually a self-limiting disease; however, it may cause significant morbidity especially in the elderly. It is more common in older people and individuals with immunocompromised conditions. Antiviral drugs can shorten the duration and the severity of the illness and need to be started as soon as possible after the appearance of the rash. Gabapentin and tricyclic antidepressant are effective in postherpetic neuralgia. Vaccine can reduce the risk of infection and its associated pain. Typically, it occurs once in a lifetime, but some individuals may have more than one episode.

## 1. Introduction

This case illustrates the importance of thorough clinical examination of patients presenting with rare or uncommon manifestations of common diseases.

## 2. Case Report 

A 67-year-old man was referred to the general medical clinic by his GP with a 3-week history of tiredness, dysuria, and frequent micturition that had not responded to antibiotics. He denied having haematuria, hesitancy, weak stream, or postmicturition dribbling. There was no fever, rigors, or loin pain. His urinalysis was normal. Urine culture, sent by his GP, was negative for infection. A routine battery of blood tests, including PSA, was normal.

On further questioning, he described penile numbness and constipation. He denied any abdominal pain, nausea, or vomiting. He also denied any weakness or sensory changes in the limbs.

He had past history of type 2 diabetes mellitus, heart failure, coronary artery bypass surgery, chronic obstructive pulmonary disease, hypothyroidism, and peripheral vascular disease.

**Figure 1:**
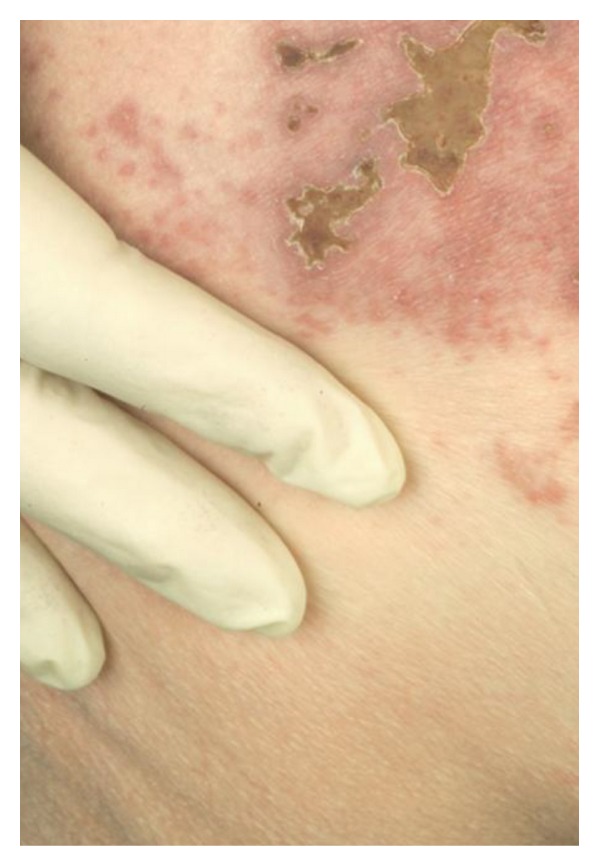


Neurological examination was unremarkable, apart from a subjective sensation of numbness of the glans penis. However, when the patient turned over to have his anal tone tested, a classic rash of herpes zoster was found on his left buttock involving S2-S3 dermatomes. The patient was totally unaware of the rash (Figure 1).

He was treated for shingles; however, two weeks later he was readmitted with a sudden onset of deafness in his left ear and poor balance. An ENT specialist saw him, and his audiogram showed a dead left ear. CT and subsequent MRI scans of the brain were normal. He was diagnosed as having acute vestibular failure secondary to herpes zoster.

## 3. Discussion

Varicella zoster virus (VZV) is associated with two distinct disease entities: chicken pox, which is primarily seen in children, and herpes zoster (shingles), which occurs predominantly in an older age group [1]. Herpes zoster (HZ) is caused by the reactivation of the VZV, which remains dormant in the geniculate and Gasserian and dorsal root ganglia following a primary chicken pox infection. It may remain latent for decades before reactivation [2]. Approximately 1% of the population develops herpes zoster in their lifetime. In the United States, the annual incidence is 2 cases per 1000 patient-years [3] increasing to 9 cases per 1000 patient-years in those above 80 years old [4]. Recurrent episodes of HZ are rare in immunocompetent individuals [5] but may be more commonly observed in immunocompromised patients. With a decline in the immunity, the virus travels down along the affected nerve causing both intraneural and perineural inflammation, which is associated with nerve cell necrosis, lymphocytic infiltration, and haemorrhage [6].

HZ results in a unilateral localised vesicular skin eruption that is usually confined to one dermatome but may involve up to three adjacent dermatomes [7]. Rarely, HZ may present purely as pain without any skin eruption, a condition called “zoster sine herpete” [8]. HZ most commonly affects the thoracolumbar (50–60%) and facial dermatomes (10–20%) [9], while involvement of sacral dermatomes is uncommon and accounts for only 5% of all cases of HZ [10]. 

Whilst it usually presents as a self-limiting vesicular eruption, it may be associated with the development of a whole host of serious complications. Early treatment of HZ may minimise the risk of some of these complications. The commonest complication is postherpetic neuralgia, which is more common in the elderly, affecting 47% of those over 60 years who develop HZ [11]. Classically, the neuralgia pain increases in severity as the day goes by, reaching a crescendo at night that interferes with sleep [12]. Other recognized complications include secondary bacterial infection; meningoencephalitis; transverse myelitis; cerebral vasculitis; pneumonitis; pancreatitis; myocarditis; hepatitis; contralateral hemiplegia resembling a stroke, which may occur weeks or even months after the initial attack; cranial nerve palsy; peripheral nerve palsy; disseminated infection in immunocompromised patients; and urinary retention. 

The urinary retention that occurs after herpes zoster infection is due to detrusor areflexia, which was first reported by Davidshah in 1890 [13]. Since then, more than 300 other cases of urogenital problems complicating herpes zoster infection have been reported in the literature [14–22]. Cystoscopy in such patients shows intravesicular lesions with surrounding inflammation of the wall of the urinary bladder [23]. Acute retention of urine is more common in women than in men [24]. In the case we report here, the patient presented with dysuria and frequent micturition rather than retention. This is probably due to detrusor muscle overactivity caused by inflammation of the bladder wall and possibly a degree of prostatitis. To our knowledge, this is the first reported case of its kind. Clason et al. [6] reported a case of granulomatous prostatitis associated with urinary retention; however, our case presented with symptoms of cystitis and not retention.

A case of erectile dysfunction and urinary retention was reported by Erol et al. [25] while our case presented with altered sensation in the glans penis (hypalgesia) due to involvement of S3 segment.

Our patient also reported constipation. Bowel problems complicating sacral HZ infection have been reported, including constipation due to dysfunction of the anal sphincter [26] and pseudo-obstruction. The exact mechanism of zoster associated pseudo-intestinal obstruction has not been fully elucidated; however, the segmental spasm and proximal dilatation may be due to the direct viral invasion of the autonomic supply of the bowel [27, 28].

HZ has been reported to cause ipsilateral deafness following ophthalmic zoster [29], and sudden profound hearing loss has also been shown to complicate cases of Ramsay Hunt syndrome [30, 31]. Our patient experienced sudden deafness, but his HZ infection was remote from both of his ears. The other unusual feature of our case was the absence of any pain at the site of the lesion. The patient was totally unaware of the shingles. Perhaps the genitourinary symptoms masked the pain of the zoster lesions.

## References

[1] Carbone V, Leonardi A, Pavese M, Raviola E, Giordano M (2004). Herpes zoster of the trigeminal nerve: a case report and review of the literature. *Minerva Stomatologica*.

[2] Arvin AM (1996). Varicella-zoster virus. *Clinical Microbiology Reviews*.

[3] Gudmundsson S, Helgason S, Sigurdsson JA (1996). The clinical course of herpes zoster: a prospective study in primary care. *European Journal of General Practice*.

[4] Insinga RP, Itzler RF, Pellissier JM, Saddier P, Nikas AA (2005). The incidence of herpes zoster in a United States administrative database. *Journal of General Internal Medicine*.

[5] Hope-Simpson RE (1965). The nature of herpes zoster: along term study and a new hypothesis. *Proceedings of the Royal Society of Medicine*.

[6] Clason AE, McGeorge A, Garland C, Abel BJ (1982). Urinary retention and granulomatous prostatitis following sacral Herpes zoster infection. A report of 2 cases with a review of the literature. *The British Journal of Urology*.

[7] Weaver BA (2007). The burden of herpes zoster and postherpetic neuralgia in the United States. *Journal of the American Osteopathic Association*.

[8] Dworkin RH, Portenoy RK (1996). Pain and its persistence in herpes zoster. *Pain*.

[9] Spray A, Glaser DA (2002). Herpes zoster of the penis: an unusual location for a common eruption. *Journal of the American Academy of Dermatology*.

[10] Oates JK, Greenhouse PRDH (1978). Retention of urine in anogenital herpetic infection. *The Lancet*.

[11] Kost RG, Straus SE (1996). Postherpetic neuralgia—pathogenesis, treatment, and prevention. *The New England Journal of Medicine*.

[12] Odrcich M, Bailey JM, Cahill CM, Gilron I (2006). Chronobiological characteristics of painful diabetic neuropathy and postherpetic neuralgia: diurnal pain variation and effects of analgesic therapy. *Pain*.

[13] Davidshah E (1890). Communication. *Berliner und Münchener Tierärztliche Wochenschrift*.

[14] Broseta E, Osca JM, Morera J, Martinez-Agullo E, Jimenez-Cruz JF (1993). Urological manifestations of herpes zoster. *European Urology*.

[15] Yamanishi T, Yasuda K, Sakakibara R (1998). Urinary retention due to herpes zoster virus infection. *Neurourology and Urodynamics*.

[16] Izumi AK, Edwards J (1973). Herpes zoster and neurogenic bladder dysfunction. *The Journal of the American Medical Association*.

[17] Rankin JT, Sutton RA (1969). Herpes zoster causing retention of urine. *The British Journal of Urology*.

[18] Rothrock JF, Walicke PA, Swenson MR (1986). Neurogenic bladder from occult herpes zoster. *Postgraduate Medicine*.

[19] Acheson J, Mudd D (2004). Acute urinary retention attributable to sacral herpes zoster. *Emergency Medicine Journal*.

[20] Jellink EH, Tulloch WS (1983). Herpes zoster with dysfunction of bladder and anus. *Archives of Dermatology*.

[21] Hiraga A, Nagumo K, Sakakibara R, Kojima S, Fujinawa N, Hashimoto T (2003). Loss of urinary voiding sensation due to herpes zoster. *Neurourology and Urodynamics*.

[22] Gibon NK (1956). Acase of herpes zoster with involvement of urinary bladder. *The British Journal of Urology*.

[23] Tsai HN, Wu WJ, Huang SP (2002). Herpes zoster induced neuropathic bladder—a case report. *The Kaohsiung journal of medical sciences*.

[24] Dauleh MI, Byrne DJ (1995). Unusual cause for acute retention of urine. *Journal of the Royal College of Surgeons of Edinburgh*.

[25] Erol B, Avaci A, Eken C, Ozgok Y (1976). Urinary retention, erectile dysfunction and meningitis due to sacral herpes zoster. *The Lancet*.

[26] Cohen LM, Fowler JF, Owen LG, Callen JP (1993). Urinary retention associated with herpes zoster infection. *International Journal of Dermatology*.

[27] Tribble DR, Church P, Frame JN (1993). Gastrointestinal visceral motor complications of dermatomal herpes zoster: report of two cases and review. *Clinical Infectious Diseases*.

[28] Nomdedeu JF, Nomdedeu J, Martino R (1995). Ogilvie’s syndrome from disseminated varicella-zoster infection and infarcted celiac ganglia. *Journal of Clinical Gastroenterology*.

[29] Scott MJ, Scott MJ (1983). Ipsilateral deafness and herpes zoster ophthalmicus. *Archives of Dermatology*.

[30] Hiraide F, Kakoi H, Miyoshi S, Morita M (1988). Acute profound deafness in Ramsay Hunt syndrome. Two case reports. *Acta Oto-Laryngologica, Supplement*.

[31] Adour KK (1994). Otological complications of herpes zoster. *Annals of Neurology*.

